# *Sox6* Differentially Regulates Inherited Myogenic Abilities and Muscle Fiber Types of Satellite Cells Derived from Fast- and Slow-Type Muscles

**DOI:** 10.3390/ijms231911327

**Published:** 2022-09-26

**Authors:** Zihao Zhang, Shudai Lin, Wen Luo, Tuanhui Ren, Xing Huang, Wangyu Li, Xiquan Zhang

**Affiliations:** 1 Department of Animal Genetics, Breeding and Reproduction, College of Animal Science, South China Agricultural University, Guangzhou 510642, China; 2 Guangdong Provincial Key Lab of Agro-Animal Genomics and Molecular Breeding, and Key Lab of Chicken Genetics, Breeding and Reproduction, Ministry of Agriculture, South China Agricultural University, Guangzhou 510642, China; 3 College of Coastal Agricultural Sciences, Guangdong Ocean University, Zhanjiang 524000, China; 4 Lingnan Guangdong Laboratory of Agriculture, South China Agricultural University, Guangzhou 510642, China; 5 State Key Laboratory for Conservation and Utilization of Subtropical Agro-Bioresources, South China Agricultural University, Guangzhou 510642, China

**Keywords:** *Sox6*, muscle satellite cells, muscle fiber types, developmental differences

## Abstract

Adult skeletal muscle is primarily divided into fast and slow-type muscles, which have distinct capacities for regeneration, metabolism and contractibility. Satellite cells plays an important role in adult skeletal muscle. However, the underlying mechanisms of satellite cell myogenesis are poorly understood. We previously found that *Sox6* was highly expressed in adult fast-type muscle. Therefore, we aimed to validate the satellite cell myogenesis from different muscle fiber types and investigate the regulation of *Sox6* on satellite cell myogenesis. First, we isolated satellite cells from fast- and slow-type muscles individually. We found that satellite cells derived from different muscle fiber types generated myotubes similar to their origin types. Further, we observed that cells derived from fast muscles had a higher efficiency to proliferate but lower potential to self-renew compared to the cells derived from slow muscles. Then we demonstrated that *Sox6* facilitated the development of satellite cells-derived myotubes toward their inherent muscle fiber types. We revealed that higher expression of Nfix during the differentiation of fast-type muscle-derived myogenic cells inhibited the transcription of slow-type isoforms (*MyH7B*, *Tnnc1*) by binding to *Sox6*. On the other hand, *Sox6* activated *Mef2C* to promote the slow fiber formation in slow-type muscle-derived myogenic cells with *Nfix* low expression, showing a different effect of *Sox6* on the regulation of satellite cell development. Our findings demonstrated that satellite cells, the myogenic progenitor cells, tend to develop towards the fiber type similar to where they originated. The expression of *Sox6* and *Nfix* partially explain the developmental differences of myogenic cells derived from fast- and slow-type muscles.

## 1. Introduction

Most mammalian skeletal muscle fibers are classified into four myosin heavy chain (MyHC) isoforms (slow type I, oxidative type IIA, fast type IIB and intermediate type IID/X) and are divided into two muscle fiber types (the slow type, which is red, oxidative and resistant to fatigue, and the fast type, which is white, glycolytic and fatigable [1]), based on their proportion of the isoforms, advantage of contraction speed or endurance and dominant metabolic way [1,2]. Adult skeletal muscle has a remarkable capability for regeneration following muscle damage mainly due to the function of satellite cells, one of the myogenic progenitors localized between the muscle fiber membrane and the basal lamina [3]. Muscle satellite cells, are myogenic stem cells, which play an essential role in muscle regeneration and hypertrophy by differentiating into myofibers and contributing to myonuclear accretion [3,4]. Number maintenance and functional exertion of satellite cells are essential for muscle regeneration ability and body homeostasis [5].

Many studies have reported the contribution of satellite cells to skeletal muscle maturation, regeneration, health, disease, aging and exercise adaptation in various species [6]. Moreover, satellite cells were also found to be required for hypertrophic growth in young adults [7]. Previous research reported that *Pax7*(+) cells required for fetal myogenesis [8] and adult myogenesis (muscle growth, fiber maturation and regeneration) are mediated by adult myogenic progenitors [9,10]. Further, abnormal small fibers and markedly decreased muscle mass with few myo-nuclei were reported in skeletal muscle of mice surviving the deletion of *Pax7* [11]. These studies highlighted the crucial role of satellite cells in adult myogenesis. A recent discovery showed that satellite cells possess a high population heterogeneity in homeostasis, aging and disease [6]. Moreover, satellite cells derived from fast- or slow-type muscles are functionally heterogeneous cells with different cell proliferation and differentiation potentials [12,13]. It has been reported that the fate of satellite cells during muscle regeneration is governed by a complex network of intrinsic and extrinsic regulators [6,14]. However, the underlying molecular mechanisms of these intrinsic regulations are not well understood.

In poultry, the pectoral muscle (PM) is generally considered to be a fast-type muscle and the leg muscle (LM) to be a slow-type muscle [15,16]. Immunofluorescence staining and western blot with specific myosin heavy chain isoforms antibodies indicate that the chicken PM is mainly composed of fast-type fibers and LM is mainly composed of slow-type fibers [17,18]. Our previous work found that SRY-box 6 (*Sox6*) transcription factor was highly expressed in the adult chicken fast-type muscle PM and had a regulatory role in muscle-derived myoblasts [19]. *Sox6* plays a critical role in the regulation of skeletal muscle fiber types [20]. However, little is known about the function of *Sox6* in satellite cell development and whether *Sox6* contributes to the postnatal muscle fiber type regulation by regulating satellite cell development.

In this study, we isolated satellite cells from fast-type PM and slow-type LM separately. We observed that myoblasts from PM-derived satellite cells (PM-MBs) and myoblasts from LM-derived satellite cells (LM-MBs) tended to develop towards their origins. Concerning the role of *Sox6* in satellite cells development, *Sox6* regulated the myogenesis of the satellite cells, directly promoting the expression of fast-type fiber isoforms (*MyH1A*, *Tnnc2*, *Tnni2* and *Tnnt3*). In particular, during myogenic differentiation, the differential expressions of *Nfix* between myoblasts derived from fast-type and slow-type muscles led to the different roles of *Sox6* in slow-type muscles-derived myoblasts. These observations partially explained the myogenic functional differences between the satellite cells derived from fast-type and slow-type muscles, indicating the essential roles of *Sox6* in the regulation of myogenic cells.

## 2. Results

### 2.1. Different Myogenic Potentials in Satellite Cells of Distinct Muscle Fiber Types

**Figure 1:**
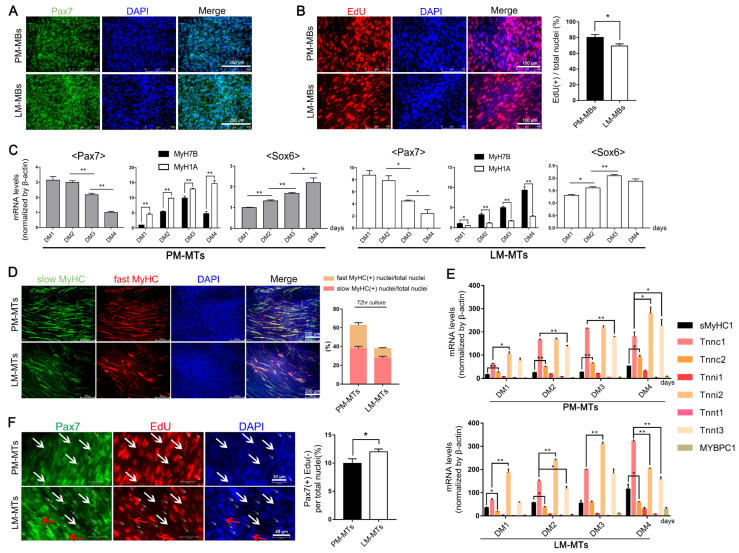
Differences in the myogenic capacity of the satellite cells from fast- and slow-type muscles. (**A**) Freshly isolated satellite cells from PM and LM were stained with satellite cell marker Pax7 (green) and DAPI (blue). (**B**) Satellite cells isolated from PM and LM were cultured in the growth medium with EdU for 6 h. The proliferating cells were stained with Edu (red) and nuclei were stained with DAPI (blue) (mean ± SEM; * *p* < 0.05; n = 3; two-tailed Student’s *t*-test). (**C**) PM-MBs and LM-MBs were cultured in DM for 4 days, the relative expression levels of *Pax7*, *MyH1A*, *MyH7B* and *Sox6* in myotubes in DM in different days were quantified by qPCR (mean ± SEM; * *p* < 0.05; ** *p* < 0.01; n = 3; two-tailed Student’s *t*-test or one-way analysis of variance with Tukey’s multiple comparison test). (**D**) After inducing differentiation for 3 days, myotubes in PM-MTs and LM-MTs were performing double immunofluorescence staining with S58 (slow-type MyHC, green) and F59 (fast-type MyHC, red) and nuclei were stained with DAPI (blue). The slow MyHC(+) nuclei or fast MyHC(+) nuclei were counted and the proportion of slow MyHC(+) cells or fast MyHC(+) cells relative to the total nuclei was quantified (mean ± SEM; n = 3). (**E**) The relative expression levels of the slow-type fiber isoforms *sMyHC1*, *Tnnc1*, *Tnni1*, *Tnnt1*, *MYBPC1* and the fast-type fiber isoforms *Tnnc2*, *Tnni2*, *Tnnt3* in PM-MTs and LM-MTs in DM in different days were quantified by qPCR (mean ± SEM; * *p* < 0.05; ** *p* < 0.01; n = 3; one-way analysis of variance with Tukey’s multiple comparison test). (**F**) PM-MBs and LM-MBs were cultured in DM for 5 days and EdU was added to the culture medium 24 h prior to harvest. In the end, the cells were stained with Pax7 (green) and EdU (red) (mean ± SEM; * *p* < 0.05; n = 3; two-tailed Student’s *t*-test). White arrows represent part of the typical Pax7(+)EdU(−) cells and red arrows represent part of the typical Pax7(+)EdU(+) cells.

We isolated satellite cells from PM and LM to validate the myogenic differences between satellite cells derived from slow- and fast-type muscles (Figure 1A). Edu staining demonstrated that there was a higher number of EdU(+) cells in PM-MBs compared to LM-MBs (Figure 1B), indicating that the PM-MBs proliferated faster than the LM-MBs. Quiescent satellite cells continuously express *Pax7* and down-regulate *Pax7* when activated [21]. Here, the expression levels of *Pax7* gradually declined in differentiation medium (DM), indicating that the satellite cells exited the quiescent state and differentiated into myotubes (Figure 1C). According to the qPCR results, with cell differentiation, the expression of *MyH1A* (myosin, heavy chain 1A) was significantly higher than *MyH7B* (myosin heavy chain 7B) in myotubes formed from PM-MBs (PM-MTs) (Figure 1C). In contrast, the myotubes formed from LM-MB (LM-MTs) highly expressed *MyH7B* (Figure 1C). Double immunofluorescence staining showed that slow MyHC (MyH7B)(+) fibers were in a higher proportion than fast MyHC (MyH1A)(+) fibers in LM-MTs (Figure 1D). In addition, the ratio of the fast MyHC(+) fibers to slow MyHC(+) fibers in PM-MTs was higher than that in LM-MTs (Figure 1D). Furthermore, qPCR results indicated that PM-MTs highly expressed fast-type isoforms *Tnni2* (troponin I2,) and *Tnnt3* (troponin T3), while the expression of slow-type isoforms *sMyHC1* (slow myosin heavy chain 1), *Tnni1* (troponin I1) and *Tnnt1* (troponin T1) were low (Figure 1E). As the differentiation proceeded, the expression of slow-type isoform *Tnnc1* (troponin C1) increased rapidly and exceeded the *Tnni2* and *Tnnt3* in LM-MTs (Figure 1E). Therefore, the satellite cells intrinsically tended to differentiate into specific fiber types of the muscle from which they derived.

When quiescent satellite cells were activated, the cells down-regulate *Pax7*, then the satellite cell-derived myogenic cells (myoblasts) enter the cell cycle and undergo limited proliferation, most of them differentiating into myotubes [22]. The rest exit the cell cycle, highly re-express *Pax7*, remain in the G0-phase and no longer proliferate and differentiate, entering back to the quiescent state to maintain the satellite cell pool and completing the cell self-renewal [22]. We cultured PM-MBs and LM-MBs in the DM for 5 days to investigate the retention number and cell cycle status of satellite cells in the later stage of cell differentiation. We detected Pax7(+)EdU(+) cells (proliferating satellite cells), Pax7(−)EdU(+) cells (cells that had been activated to proliferate and differentiate) and Pax7(+)EdU(−) cells (satellite cells entered back to the quiescent state or maintained in G0-phase) in PM-MTs and LM-MTs. We found that the number of Pax7(+)EdU(−) cells was higher in LM-MTs than in PM-MTs (Figure 1F), implying that more satellite cells stay or return to the quiescent stage in LM-MTs and the satellite cells derived from slow-type LM possessed a higher self-renewal potential.

### 2.2. Sox6 Promotes the Myogenesis and Development of Satellite Cells Derived from Fast Type-Enriched Pectoral Muscles towards Intrinsic Tendency

**Figure 2:**
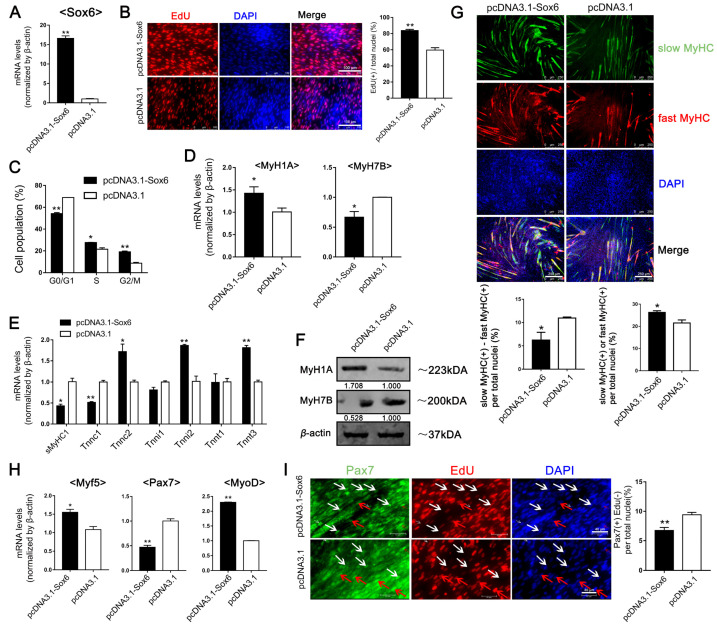
*Sox6* in PM-derived satellite cell promotes cell proliferation and fast-type fiber formation, but decreases slow-type fiber formation and cell self-renewal potential. (**A**) The relative expression levels of *Sox6* in *Sox6*-overexpressing PM-MBs were quantified by qPCR (mean ± SEM; ** *p* < 0.01; n = 3; two-tailed Student’s *t*-test). (**B**) Proliferation of *Sox6*-overexpressing PM-MBs were assessed by Edu (mean ± SEM; ** *p* < 0.01; n = 3; two-tailed Student’s *t*-test). (**C**) Cell cycle analysis of *Sox6*-overexpressing PM-MBs (mean ± SEM; * *p* < 0.05; ** *p* < 0.01; n = 3; two-tailed Student’s *t*-test). (**D**) After inducing differentiation for 3 days, the relative expression levels of *MyH1A* and *MyH7B* in *Sox6*-overexpressing PM-MTs were quantified by qPCR (mean ± SEM; * *p* < 0.05; n = 3; two-tailed Student’s *t*-test). (**E**) After inducing differentiation for 3 days, the relative expression levels of fast- and slow-type fiber isoforms in *Sox6*-overexpressing PM-MTs were quantified by qPCR (mean ± SEM; * *p* < 0.05; ** *p* < 0.01; n = 3; two-tailed Student’s *t*-test). (**F**) Western blot on lysates from *Sox6*-overexpressing PM-MTs and control groups myotubes after inducing differentiation for 3 days. *β*-actin was used to normalize. (**G**) After inducing differentiation for 3 days, myotubes derived from *Sox6*-overexpressing PM-MTs were stained against S58 (slow-type MyHC, green) and F59 (fast-type MyHC, red) and nuclei were counterstained with DAPI. The slow MyHC(+) nuclei or fast MyHC(+) nuclei were counted. The proportion of slow MyHC(+) cells − fast MyHC(+) cells among total nuclei and the proportion of cells in slow MyHC(+) myotubes or fast MyHC(+) myotubes among total nuclei are presented as mean ± SEM (* *p* < 0.05; n = 3; two-tailed Student’s *t*-test). (**H**) After inducing differentiation for 3 days, the relative expression levels of *Myf5*, *Pax7* and *MyoD* in *Sox6*-overexpressing PM-MBs were quantified by qPCR (mean ± SEM; * *p* < 0.05; ** *p* < 0.01; n = 3; two-tailed Student’s *t*-test). (**I**) *Sox6*-overexpressing PM-MBs were cultured in DM for 5 days and EdU was added to the culture medium 24 h prior to harvest. In the end, the cells were stained against Pax7 (green) and EdU (red) and the numbers were counted (mean ± SEM; ** *p* < 0.01; n = 3; two-tailed Student’s *t*-test). White arrows represented part of the typical Pax7(+)EdU(−) cells and red arrows represented part of the typical Pax7(+)EdU(+) cells.

Our previous study found that *Sox6* was highly expressed in adult chicken PM; here, we found that the expression of *Sox6* in PM-MTs gradually increased during the differentiation. However, the *Sox6* expression in LM-MTs peaked at DM day 3 and then slightly declined (Figure 1C). Then we overexpressed or suppressed *Sox6* in PM-MBs to assess its effect (Figure 2A and Figure S1A). Proliferation assay and cell cycle analysis showed that *Sox6* overexpression significantly promoted the PM-MB proliferation (Figure 2B,C), whereas *Sox6* inhibition significantly downregulated the PM-MB proliferation (Figure S1B,C).

After inducing the differentiation, *Sox6* overexpression and inhibition was performed in PM-MTs. qPCR results revealed that *Sox6* overexpression significantly increased the expression of *MyH1A* and other fast-type isoforms (*Tnnc2*, *Tnni2*, *Tnnt3*), while attenuated *MyH7B* and other slow-type isoforms’ (*sMyHC1*, *Tnnc1*) expression in PM-MTs (Figure 2D,E). In contrast, inhibition of *Sox6* in PM-MTs showed reduced expression of *MyH1A*, *Tnnc2*, *Tnni2* and *Tnnt3*, whereas the expression of *MyH7B*, *sMyHC1*, *Tnnc1* and *MYBPC1* were increased (Figure S1D,E). Similar to the qPCR results, western blotting demonstrated increased MyH1A and decreased MyH7B protein levels in *Sox6*-overexpressing PM-MTs compared to control (Figure 2F). Inhibiting *Sox6* in PM-MTs decreased MyH1A and increased MyH7B protein levels (Figure S1F). To obtain a further validation, we used double immunofluorescence staining on the myotubes. Results showed that *Sox6* overexpression significantly reduced the disparity in the proportion between slow (MyH7B) and fast (MyH1A) MyHC(+) myotubes in PM-MTs (Figure 2G), supporting the above finding that *Sox6* increased the fast-type fibers and decreased the slow-type fibers in PM-MTs. In contrast, suppression of *Sox6* in PM-MTs significantly increased the disparity in the proportion between slow and fast MyHC(+) myotubes (Figure S1G).

In addition, the myogenic differentiation (all types of MyHC(+) per total nuclei) was significantly improved in *Sox6*-overexpressing PM-MTs, while inhibition of *Sox6* reduced the myogenic differentiation (Figure 2G and Figure S1G). Besides, qPCR results also demonstrated significantly increased expression of *MyoD* (myogenic differentiation 1), *Myf5* (myogenic factor 5) and down-regulation of *Pax7*, which regulate the myogenic cell differentiation [23], in *Sox6*-overexpressing PM-MTs (Figure 2H). The opposite result was revealed in *Sox6*-inhibited PM-MTs (Figure S1H). Further, we investigated the retention number and cell cycle status of satellite cells after 5 days of cell differentiation. We found that, the *Sox6*-overexpressing PM-MTs contained a significantly lower number of Pax7(+)EdU(−) cells and *Sox6*-inhibited PM-MTs contained an increased number of Pax7(+)EdU(−) cells (Figure 2I and Figure S1I). The reduction in the number of Pax7(+)EdU(−) cells in *Sox6*-overexpressing PM-MTs reflected a diminished cell self-renewal potential.

### 2.3. Different Roles of Sox6 in Satellite Cells Derived from Slow Type-Enriched Leg Muscles

**Figure 3:**
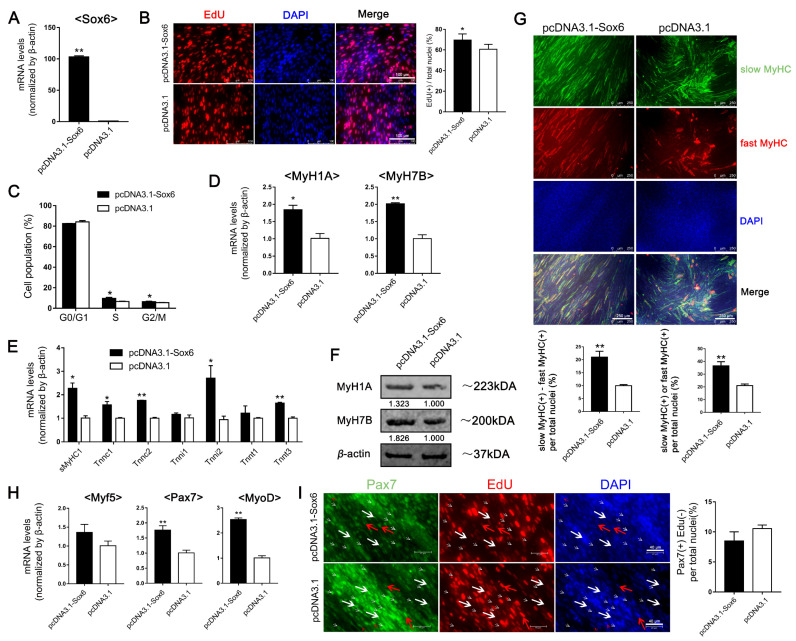
*Sox6* in LM-derived satellite cells promotes cell proliferation, fast-type and slow-type fibers’ formation. (**A**) The relative expression levels of *Sox6* in *Sox6*-overexpressing LM-MBs were quantified by qPCR (mean ± SEM; ** *p* < 0.01; n = 3; two-tailed Student’s *t*-test). (**B**) Proliferation of *Sox6*-overexpressing LM-MBs were assessed by Edu (mean ± SEM; * *p* < 0.05; n = 3; two-tailed Student’s t-test). (**C**) Cell cycle analysis of *Sox6*-overexpressing LM-MBs (mean ± SEM; * *p* < 0.05; n = 3; two-tailed Student’s *t*-test). (**D**) After inducing differentiation for 3 days, the relative expression levels of *MyH1A* and *MyH7B* in *Sox6*-overexpressing LM-MTs were quantified by qPCR (mean ± SEM; * *p* < 0.05; ** *p* < 0.01; n = 3; two-tailed Student’s *t*-test). (**E**) After inducing differentiation for 3 days, the relative expression levels of fast- and slow-type fiber isoforms in *Sox6*-overexpressing LM-MTs were quantified by qPCR (mean ± SEM; * *p* < 0.05; ** *p* < 0.01; n = 3; two-tailed Student’s *t*-test). (**F**) Western blot on lysates from *Sox6*-overexpressing LM-MTs and control groups myotubes after inducing differentiation for 3 days. *β*-actin was used to normalize. (**G**) After inducing differentiation for 3 days, myotubes derived from *Sox6*-overexpressing LM-MTs were stained against S58 (slow-type MyHC, green) and F59 (fast-type MyHC, red) and nuclei were counterstained with DAPI. The slow MyHC(+) nuclei or fast MyHC(+) nuclei were counted. The proportion of slow MyHC(+) cells − fast MyHC(+) cells among total nuclei and the proportion of cells in slow MyHC(+) myotubes or fast MyHC(+) myotubes among total nuclei are presented as mean ± SEM (** *p* < 0.01; n = 3; two-tailed Student’s *t*-test). (**H**) After inducing differentiation for 3 days, the relative expression levels of *Myf5*, *Pax7* and *MyoD* in *Sox6*-overexpressing LM-MBs were quantified by qPCR (mean ± SEM; ** *p* < 0.01; n = 3; two-tailed Student’s *t*-test). (**I**) *Sox6*-overexpressing LM-MBs were cultured in DM for 5 days and EdU was added to the culture medium 24 h prior to harvest. In the end, the cells were stained against Pax7 (green) and EdU (red) and the numbers were counted (mean ± SEM; n = 3; two-tailed Student’s t-test). White arrows represented part of the typical Pax7(+)EdU(−) cells and red arrows represented part of the typical Pax7(+)EdU(+) cells.

Considering the differential myogenic potentials of satellite cell-derived myoblasts from PM and LM, we overexpressed or suppressed *Sox6* to detect the effect of *Sox6* on LM-MBs (Figure 3A and Figure S2A). Proliferation assay and cell cycle analysis revealed that *Sox6* could improve the proliferation of LM-MBs, while inhibition of *Sox6* attenuated the proliferation (Figure 3B,C and Figure S2B,C). After inducing the differentiation, *Sox6* overexpression and inhibition was performed in LM-MTs. qPCR results revealed that overexpression of *Sox6* expectedly improved the expression of *MyH1A*, *Tnnc2*, *Tnni2* and *Tnnt3* in LM-MTs (Figure 3D,E). However, overexpression of *Sox6* in LM-MTs unexpectedly increased the expression of *MyH7B*, *sMyHC1* and *Tnnc1* (Figure 3D,E). On the contrary, the expression of *MyH1A*, *MyH7B*, *sMyHC1*, *Tnnc1, Tnnc2*, *Tnni2* and *Tnnt3* were reduced in *Sox6*-inhibited LM-MTs (Figure S2D,E). Western blotting results showed increased protein levels of MyH1A and MyH7B in *Sox6*-overexpressing LM-MTs compared to the control (Figure 3F), while inhibition of *Sox6* downregulated the expression of MyH1A and MyH7B in LM-MTs (Figure S2F). Double immunofluorescence staining showed that *Sox6*-overexpressing LM-MTs significantly increased the disparity in the proportion between slow and fast MyHC(+) myotubes (Figure 3G). In contrast, *Sox6*-inhibited LM-MTs hardly decreased the disparity between slow and fast MyHC(+) myotubes (Figure S2G). These results indicated a differential regulation of *Sox6* on muscle fiber types of the myotubes derived from PM-MBs and LM-MBs. Overexpression of *Sox6* promoted the myogenic differentiation of LM-MTs, while inhibition of *Sox6* in LM-MTs reduced the myogenic differentiation (Figure 3G,H and Figure S2G,H). Five days after cell differentiation, the number of Pax7(+)EdU(−) cells was decreased in *Sox6*-overexpressing LM-MTs and increased in *Sox6*-inhibited LM-MTs (Figure 3I and Figure S2I). However, the result was not significant.

### 2.4. Differential Expression of Nfix between LM-MBs and PM-MBs at the Differentiation Stage Leads to Different Roles of Sox6 in the Regulation of Slow-Type Fibers

**Figure 4:**
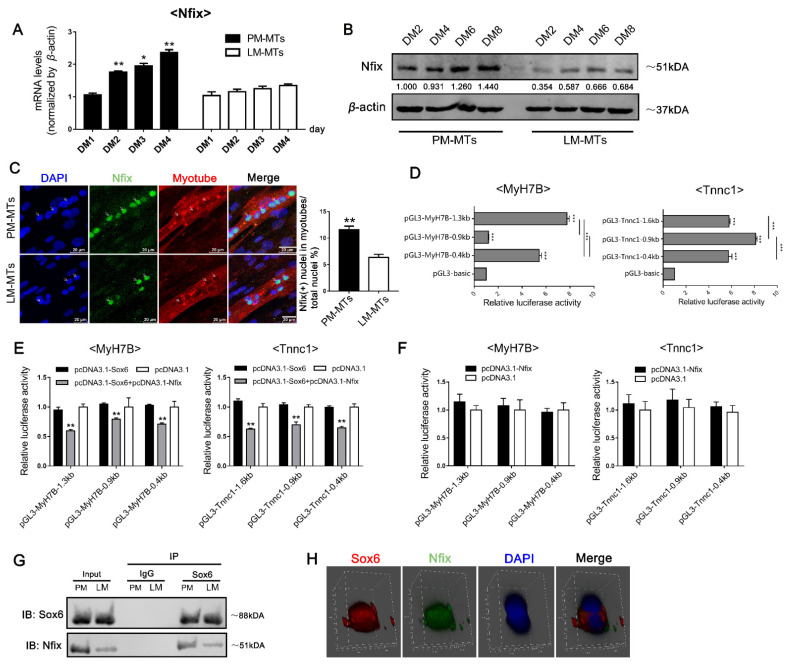
Differential expression patterns of *Nfix* between PM-MTs and LM-MTs modulate the *Sox6* down-regulation of slow-type fibers. (**A**) The relative expression levels of *Nfix* in PM-MTs and LM-MTs in DM for different days were quantified by qPCR (mean ± SEM; * *p* < 0.05; ** *p* < 0.01; n = 3; one-way analysis of variance with Tukey’s multiple comparison test). (**B**) Western blot on lysates from myotubes of PM-MTs and LM-MTs in DM for different days. *β*-actin was used to normalize. (**C**) After inducing differentiation for 5 days, myotubes derived from PM and LM were performing double immunofluorescence staining against Nfix (green) and MyHC (red) and nuclei were counterstained with DAPI (mean ± SEM; ** *p* < 0.01; n = 3; two-tailed Student’s *t*-test). (**D**) Dual-Luciferase report assay on DF-1 transfected with reporter vectors containing different lengths of 5′-upstream region of *MyH7B* and *Tnnc1* (mean ± SEM; *** *p* < 0.001; n = 6; one-way analysis of variance with Tukey’s multiple comparison test). (**E**) Dual-Luciferase report assay on DF-1 transfected with reporter vectors containing different lengths of 5′-upstream region of *MyH7B* and *Tnnc1* after overexpressing *Sox6* or *Sox6* and *Nfix* (mean ± SEM; ** *p* < 0.01; n = 6; one-way analysis of variance with Tukey’s multiple comparison test). (**F**) Dual-Luciferase report assay on DF-1 transfected with reporter vectors containing different lengths of 5′-upstream region of *MyH7B* and *Tnnc1* after overexpressing *Nfix* (mean ± SEM; n = 6; two-tailed Student’s *t*-test). (**G**) Lysates of PM-MTs and LM-MTs overexpressing *Sox6* were immunoprecipitated with *Sox6* antibody and then western blotted with Nfix antibody. IgG, negative control; IP, immunoprecipitated; IB, immunoblotting. (**H**) Myotubes derived from PM-MTs were performing double immunofluorescence staining against *Sox6* (red) and Nfix (green). The three-dimensional view of *Sox6* and Nfix localization in PM-MTs was presented through confocal microscopy.

In order to identify the possible mechanism by which *Sox6* differentially regulates the transcription of slow-type isoforms, we focused on the transcription factor *Nfix*. *Nfix*, a known regulator for the slow twitching fibers [24], had been found to influence the role of *Sox6* [25]. First, we examined *Nfix* expression in differentiating PM-MTs and LM-MTs. qPCR results showed that the expression of *Nfix* significantly upregulated with the differentiation in PM-MTs (Figure 4A), while the differences of *Nfix* expression in LM-MTs was not significant with myogenic differentiation. Further validation on protein levels demonstrated that the Nfix expression upregulated with the PM-MTs differentiation (Figure 4B). While Nfix also expressed in LM-MTs and upregulated with myogenic differentiation, the expression level was relatively low and the increase was limited (Figure 4B). Further, confocal microscopy results showed that, the PM-MTs in differentiating contained more Nfix(+) cells than LM-MTs (Figure 4C).

To further elucidate the mechanisms between *Nfix* and *Sox6* in the regulation of slow-type fibers, we performed dual-luciferase assays on DF-1 cells, non-skeletal muscle-derived cells, transfected with reporter vectors containing different lengths of 5′-upstream region of slow-type isoform genes (Figure 4D). There was no luciferase activity difference of *MyH7B* and *Tnnc1* in *Sox6*-overexpressing DF-1 compared to the control (Figure 4E). However, with the co-overexpression of *Nfix* and *Sox6*, the luciferase activities of *MyH7B* and *Tnnc1* were significantly down-regulated (Figure 4E). To further validate whether the suppressive effect of the slow-type isoforms comes from *Nfix*, we investigated the luciferase activities of *MyH7B* and *Tnnc1* under the overexpression of *Nfix* in DF-1. However, no significant change was detected (Figure 4F). Our results indicated that neither *Sox6* nor *Nfix* alone could directly affect the transcriptions of *MyH7B* and *Tnnc1*, but *Sox6* could suppress the transcriptions of slow-type isoforms by acting on their proximal promoters in a Nfix-dependent way (Figure 4D,E). Further, we performed a co-immunoprecipitation (Co-IP) assay for *Sox6* in PM-MTs and LM-MTs after overexpressing *Sox6*. Co-IP results revealed that *Sox6* could bind to Nfix in PM-MTs and LM-MTs (Figure 4G). Three-dimensional stereogram through confocal microscopy also showed a co-localization pattern between the proteins of *Sox6* and Nfix, while myo-nuclei were fusing at this location (Figure 4H and Figure S3). These results indicated that high expression of Nfix in differentiating PM-MTs caused *Sox6* and Nfix protein complex formation, exerting the suppression effect of *Sox6* on slow-type fibers.

### 2.5. Sox6 Indirectlyupregulates the Slow-Type Isoforms through the Activation of Mef2C

**Figure 5:**
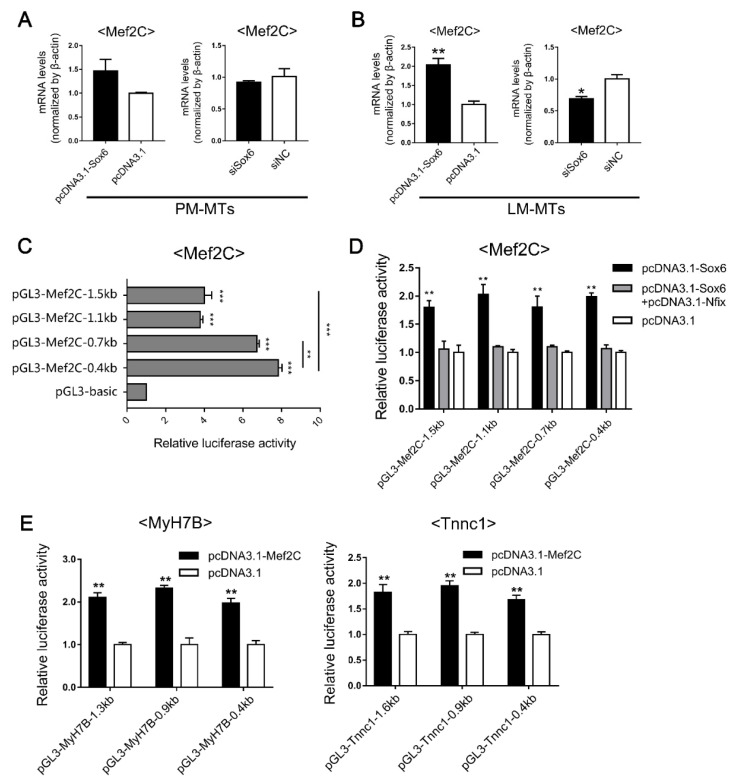
*Sox6* indirectly improves the slow-type isoforms through the activation of *Mef2C*. (**A**) After inducing differentiation for 3 days, the relative expression levels of *Mef2C* in PM-MTs or (**B**) LM-MTs after overexpression or inhibition of *Sox6* were quantified by qPCR (mean ± SEM; * *p* < 0.05; ** *p* < 0.01; n = 3; two-tailed Student’s *t*-test). (**C**) Dual-Luciferase report assay on DF-1 transfected with reporter vectors containing different lengths of 5′-upstream region of *Mef2C* (mean ± SEM; ** *p* < 0.01; *** *p* < 0.001; n = 6; one-way analysis of variance with Tukey’s multiple comparison test). (**D**) Dual-Luciferase report assay on DF-1 overexpressing *Sox6* and *Nfix* and transfected with reporter vectors containing different lengths of *Mef2C* 5′-upstream region (mean ± SEM; ** *p* < 0.01; n = 6; one-way analysis of variance with Tukey’s multiple comparison test). (**E**) Dual-Luciferase report assay on DF-1 overexpressing *Mef2C* and transfected with reporter vectors containing different lengths of 5′-upstream region of *MyH7B* and *Tnnc1* (mean ± SEM; ** *p* < 0.01; n = 6; two-tailed Student’s *t*-test).

Since *Sox6* alone did not directly bind to the promoters of *MyH7B* and *Tnnc1* (Figure 4E), implying the promoted effect of *Sox6* on slow-type isoforms in LM-MTs might be exerted through other factors. We focused on a transcription regulator *Mef2C*, which has been found to up-regulate the slow-type fibers [26,27]. According to previous research, the regulation of *Mef2C* through *Sox6* could be influenced by *Nfix* [25]. Therefore, we investigated the relationship between *Sox6*, *Nfix* and *Mef2C* in satellite cells. After inducing the myoblasts differentiation, we overexpressed and suppressed *Sox6* in PM-MTs and LM-MTs separately. We found that *Mef2C* expression was up-regulated in *Sox6*-overexpressing PM-MTs, but the effect was not significant (Figure 5A). Inhibition of *Sox6* in PM-MTs did not influence the expression of *Mef2C* (Figure 5A). However, the expression of *Mef2C* was significantly increased in *Sox6*-overexpressing LM-MTs and suppression of *Sox6* in LM-MTs significantly reduced the expression of *Mef2C* (Figure 5B).

Further investigation using dual-luciferase assays on DF-1 cells transfected with reporter vectors containing different lengths of 5′-upstream region of *Mef2C* (Figure 5C). Dual-luciferase assays showed that significantly increased luciferase activity in all *Mef2C* reporter vectors in *Sox6*-overexpressed cells (Figure 5D). However, *Sox6* overexpression did not change the luciferase activities of all reporter vectors under the co-overexpression with *Nfix* (Figure 5D). These results indicated that *Sox6* directly promoted the transcription of *Mef2C* by acting on the proximal promoter region (Figure 5C,D), but *Nfix* could interrupt the promotion. Further, we observed significant increases of luciferase activities in all slow-type isoform gene reporter vectors in DF-1 overexpressing *Mef2C* (Figure 5E), indicating that *Mef2C* could directly act on the proximal promoters of *MyH7B* and *Tnnc1* to promote the transcriptions. Given the differential *Nfix* expression between PM-MTs and LM-MTs, these results indicated that high expression of *Nfix* in PM-MTs bind to *Sox6* to hinder its promotion of *Mef2C*, while the expression of *Nfix* in LM-MTs is insufficient to exert the same effect.

### 2.6. Sox6 Decreases Slow-Type Isoforms through Binding with Nfix in PM-MTs or Increases Slow-Type Isoforms by Activating Mef2C in LM-MTs

**Figure 6:**
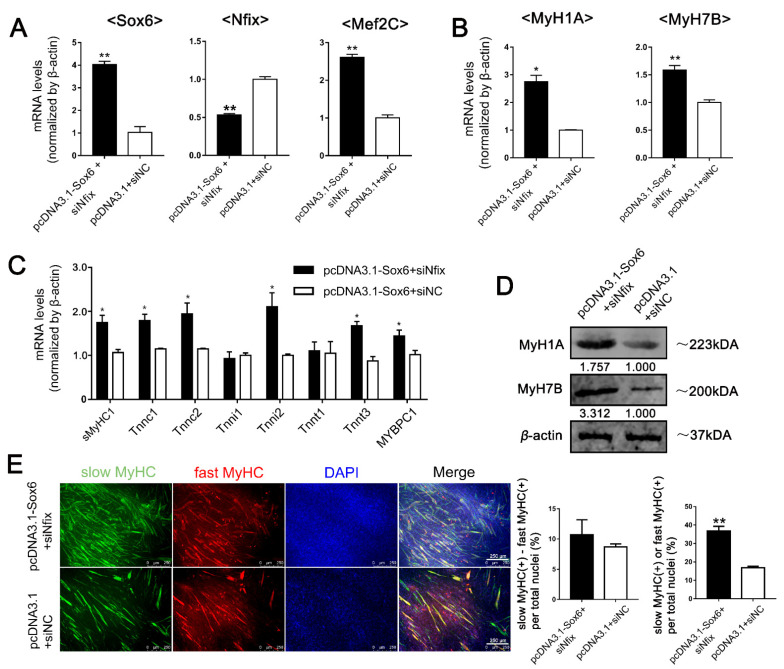
Overexpression of *Sox6* in *Nfix*-inhibited PM-MTs promotes the expression of *Mef2C* and slow-type fibers. (**A**) After inducing differentiation for 3 days, the relative expression levels of *Sox6*, *Nfix* and *Mef2C* in *Sox6*-overexpressing PM-MTs with *Nfix* inhibition were quantified by qPCR (mean ± SEM; ** *p* < 0.01; n = 3; two-tailed Student’s *t*-test). (**B**) After inducing differentiation for 3 days, the relative expression levels of *MyH1A* and *MyH7B* in *Sox6*-overexpressing PM-MTs with *Nfix* inhibition were quantified by qPCR (mean ± SEM; * *p* < 0.05; ** *p* < 0.01; n = 3; two-tailed Student’s *t*-test). (**C**) After inducing differentiation for 3 days, the relative expression levels of fast- and slow-type fiber isoforms in *Sox6*-overexpressing PM-MTs with *Nfix* inhibition were quantified by qPCR (mean ± SEM; * *p* < 0.05; n = 3; two-tailed Student’s *t*-test). (**D**) Western blot on lysates from *Sox6*-overexpressing PM-MTs with *Nfix* inhibition and control group myotubes after inducing differentiation for 3 days. *β*-actin was used to normalize. (**E**) After inducing differentiation for 3 days, myotubes derived from *Sox6*-overexpressing PM-MTs with *Nfix* inhibition were performing immunofluorescence double staining against S58 (slow-type MyHC, green) and F59 (fast-type MyHC, red) and nuclei were counterstained with DAPI. The slow MyHC(+) nuclei or fast MyHC(+) nuclei were counted. The proportion of slow MyHC(+) cells − fast MyHC(+) cells among total nuclei and the proportion of cells in slow MyHC(+) myotubes or fast MyHC(+) myotubes among total nuclei are presented as mean ± SEM (** *p* < 0.01; n = 3; two-tailed Student’s *t*-test).

To get on a further validation, we simultaneously overexpressed *Sox6* and suppressed *Nfix* in PM-MTs (Figure 6A). Expectedly, qPCR result showed a significant increase of *Mef2C* (Figure 6A), which was consistent with the effect of *Sox6* overexpression on LM-MTs (Figure 5B). In addition, we overexpressed *Mef2C* in another group of PM-MTs to validate whether its effect on slow-type isoforms was the same as the overexpression of *Sox6* in *Nfix*-inhibited PM-MTs (Figure S4A). Using qPCR, we found significantly increased expression of *MyH1A*, *MyH7B*, *sMyHC1*, *Tnnc1, Tnnc2*, *Tnni2*, *Tnnt3* and *MyBPC1* in *Sox6*-overexpressing PM-MTs with *Nfix* inhibition (Figure 6B,C). Western blotting results showed increased protein levels of MyH1A and MyH7B in *Sox6*-overexpressing PM-MTs with *Nfix* inhibition (Figure 6D). On the other hand, *Mef2C* overexpression in PM-MTs also significantly elevated the expression of *MyH7B*, *sMyHC1*, *Tnnc1* and *MyBPC1* (Figure S4B,C). Western blotting results showed increased protein levels of MyH7B in *Mef2C*-overexpressing PM-MTs (Figure S4D). Double immunofluorescence staining showed no significant increase of the disparity in the proportion between slow and fast MyHC(+) myotubes in *Sox6*-overexpressing PM-MTs with *Nfix* inhibition (Figure 6E), which was consistent with the simultaneous increases in fast and slow type fibers (Figure 6B–D). *Mef2C* overexpression significantly increased the disparity between slow and fast MyHC(+) myotubes and promoted the myogenic differentiation in PM-MTs (Figure S4E). These results indicated that *Sox6* upregulated the slow-type fibers through the promotion of *Mef2C*, but it was interrupted by *Nfix* in PM-MTs, while the fast-type fibers were improved by *Sox6*.

**Figure 7:**
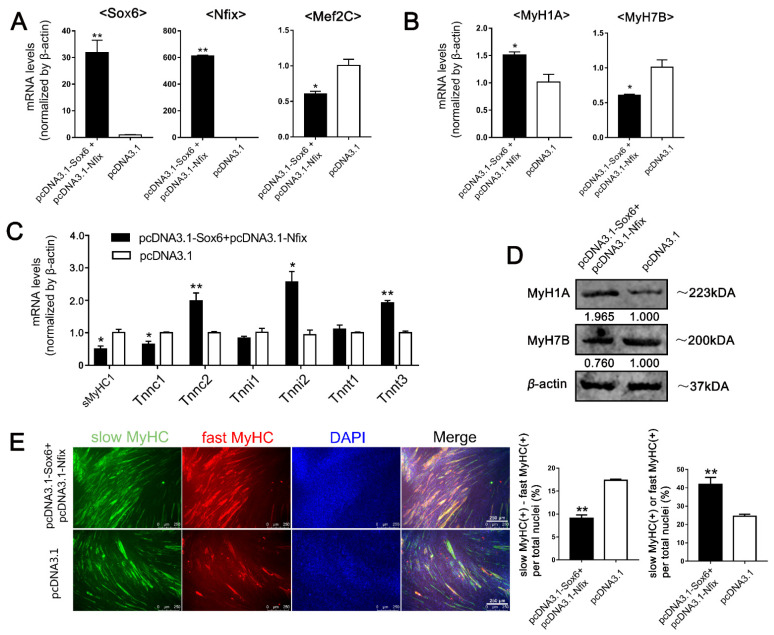
Overexpression of *Sox6* in *Nfix*-overexpressing LM-MTs inhibits the expression of *Mef2C* and slow-type fiber isoforms. (**A**) After inducing differentiation for 3 days, the relative expression levels of *Sox6*, *Nfix* and *Mef2C* in *Sox6*-overexpressing LM-MTs with *Nfix* overexpression were quantified by qPCR (mean ± SEM; * *p* < 0.05; ** *p* < 0.01; n = 3; two-tailed Student’s *t*-test). (**B**) After inducing differentiation for 3 days, the relative expression levels of *MyH1A* and *MyH7B* in *Sox6*-overexpressing LM-MTs with *Nfix* overexpression were quantified by qPCR (mean ± SEM; * *p* < 0.05; n = 3; two-tailed Student’s *t*-test). (**C**) After inducing differentiation for 3 days, the relative expression levels of fast- and slow-type fiber isoforms in *Sox6*-overexpressing LM-MTs with *Nfix* overexpression were quantified by qPCR (mean ± SEM; * *p* < 0.05; ** *p* < 0.01; n = 3; two-tailed Student’s t-test). (**D**) Western blot on lysates from *Sox6*-overexpressing LM-MTs with *Nfix* overexpression and control group myotubes after inducing differentiation for 3 days. *β*-actin was used to normalize. (**E**) After inducing differentiation for 3 days, myotubes derived from *Sox6*-overexpressing LM-MTs with *Nfix* overexpression were performing immunofluorescence double staining against S58 (slow-type MyHC, green) and F59 (fast-type MyHC, red) and nuclei were counterstained with DAPI. The slow MyHC(+) nuclei or fast MyHC(+) nuclei were counted. The proportion of slow MyHC(+) cells − fast MyHC(+) cells among total nuclei and the proportion of cells in slow MyHC(+) myotubes or fast MyHC(+) myotubes among total nuclei are presented as mean ± SEM (** *p* < 0.01; n = 3; two-tailed Student’s *t*-test).

Next, we compared the effect of *Nfix* and *Sox6* co-overexpression with the effect of *Mef2C* inhibition on LM-MTs (Figure 7A and Figure S5A). After inducing the differentiation, *Nfix*, *Sox6* co-overexpression and *Mef2C* inhibition was performed in LM-MTs separately. qPCR results showed that *Sox6* and *Nfix* co-overexpression in LM-MTs significantly increased the expression of *MyH1A*, *Tnnc2*, *Tnni2* and *Tnnt3*, while decreasing the expression of *MyH7B, sMyHC1* and *Tnnc1* (Figure 7B,C). Western blotting results demonstrated increased MyH1A and decreased MyH7B protein levels in LM-MTs with *Sox6* and *Nfix* co-overexpression (Figure 7D). Double immunofluorescence staining showed significant decrease of the disparity in the proportion between slow and fast MyHC(+) myotubes in LM-MTs with *Sox6* and *Nfix* co-overexpression (Figure 7E). These results were reminiscent of the results observed in *Sox6*-overexpressing PM-MTs (Figure 2D–G). Inhibition of *Mef2C* in LM-MTs significantly decreased the expression of *MyH7B, sMyHC1*, *Tnnc1* and *MYBPC1* (Figure S5B,C). Western blotting results showed decreased MyH7B protein levels in LM-MTs with inhibition of *Mef2C* (Figure S5D). And inhibition of *Mef2C* in LM-MTs significantly decrease of the disparity in the proportion between slow and fast MyHC(+) myotubes (Figure S5E). These results consolidated that the *Sox6* promoting effect on slow-type isoforms in LM-MTs was due to its low expression of *Nfix*, which was insufficient to hinder the promotion of *Sox6* on *Mef2C*.

**Figure 8:**
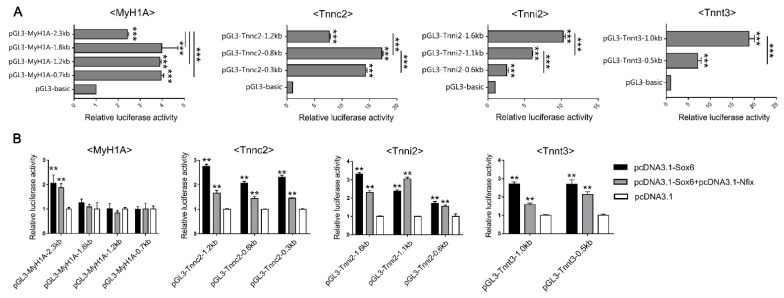
*Sox6* directly promotes the transcription of fast-type isoforms. (**A**) Dual-Luciferase report assays on DF-1 transfected with reporter vectors containing different lengths of 5′-upstream region of *MyH1A*, *Tnnc2*, *Tnni2* and *Tnnt3* (mean ± SEM; *** *p* < 0.001; n = 6; one-way analysis of variance with Tukey’s multiple comparison test). (**B**) Dual-Luciferase report assays on DF-1 overexpressing *Sox6* and *Nfix* and transfected with reporter vectors containing different lengths of 5′-upstream region of *MyH1A*, *Tnnc2*, *Tnni2* and *Tnnt3* (mean ± SEM; ** *p* < 0.01; n = 6; one-way analysis of variance with Tukey’s multiple comparison test).

In addition, we noticed that *Sox6* promoted the expression of fast-type isoforms in PM-MTs and LM-MTs with or without the presence of *Nfix*. Therefore, we performed dual-luciferase assays on DF-1 transfected with reporter vectors containing different lengths of 5′-upstream region of fast-type isoform genes (Figure 8A). Dual-luciferase assays showed increased luciferase expression in the overexpression of *Sox6* with the vector carrying the −2317 bp 5′ upstream sequence of the *MyH1A* (Figure 8B). However, the increased luciferase expression by *Sox6* was lost without the −2317 to −1823 bp 5′ upstream sequence of *MyH1A* on the vector (Figure 8B), indicating that *Sox6* directly acted on this site to enhance the *MyH1A* expression in a *Nfix*-independent way. In addition, *Sox6* alone could directly act on the proximal promoters to promote the expression of *Tnnc2*, *Tnni2* and *Tnnt3* (Figure 8A,B). These results indicated that *Sox6* independently promoted the fast-type fiber formation by directly acting on regulatory elements or proximal promoters of the fast-type isoforms.

## 3. Discussion

It was generally considered that the fiber types of muscles are primarily influenced by innervation or age and external stimulus [28,29]. However, a previous study reported that myoblasts isolated from soleus (SOL) and tibialis anterior (TA) muscles of mice retained their contractile and metabolic properties in vitro [30]. Although, compared to model species, avian body structure and genetic mechanisms are not exactly the same. However, many studies have also proved that it is a good way to study the molecular genetic regulation mechanisms using avian as model [31,32]. In the present study, we isolated the satellite cells from the fast-type muscle and slow-type muscle and found that the PM-derived cells have a faster proliferation rate. A previous review reported that multiple fiber types were generally intermingled within a single skeletal muscle, while different skeletal muscles possessed distinct proportions of fiber types instead of a single fiber type [33]. Here we found that the relative ratio of slow-type fibers was high in LM-MTs and the relative proportion of fast-type fibers in PM-MTs was highly elevated compared to LM-MTs. Thus, our study once again demonstrated that the development of muscle fiber types is regulated by the endogenous genetic mechanisms. In addition, the presence of a considerable proportion of slow-type fibers in PM-MTs might be similar to the fact that, most of the fast-type muscles in the newborn mice contain significant amounts of slow-type I fibers and the majority of them would disappear during the growth and development [34].

The self-renewal ability of satellite cells determines the capacity to restore satellite cell number [35], which is essential for the muscle regeneration and development. Rare surviving mice with a few number of satellite cells exhibited reduced body growth and marked muscle atrophy [36]. The present study implied that satellite cells derived from LM possessed a higher potential for self-renewal compared to PM. As previously reported, compared with fast-type muscle fibers, slow-type muscle fibers undergo a preferential satellite cell expansion and myonuclear addition during hypertrophy, indicating the potential differences in satellite cell requirement between different muscle fiber types [37].

Disruption of *Sox6* exons in humans with delayed speech development and attention deficit hyperactivity disorder is associated with generalized dystonic and pectus carinatum [38]. *Sox6* gene disruption in mouse resulted in growth retardation, myopathy [39]. It was shown that the *Sox6* mutant influenced the *MyHC* isoform expression patterns in myotubes formed from myoblasts in the absence of innervation [40]. Here we showed that *Sox6* could promote the PM-MB proliferation, increase the fast-type fiber formation and decrease the slow-type fiber formation in PM-MTs. A previous study demonstrated that *Sox6*-mutant fetal mice maintained slow fiber characteristics in all muscle fibers [40], indicating that *Sox6* is required for normal fast-type fiber differentiation in developing fetal skeletal muscle. Here we showed that *Sox6* could also contribute to the growth and property maintenance of fast-type muscles in adult skeletal muscle via the regulation of satellite cells. However, unlike in PM-MTs, *Sox6* led to an up-regulation of slow-type fibers in LM-MTs. Therefore, we further investigated *Nfix*, a known regulator of the *Sox6* function [25]. Recent studies reported that *Nfix* is related to skeletal muscle regeneration and satellite cell development [24,41]. Satellite cells derived from different muscles exhibited distinct gene expression profiles [12]. *Sox6* gene structure dictates its functional versatility and critical requirement for co-factors to exert its transcriptional regulation [42]. Our present study showed that *Nfix* in PM-MTs and LM-MTs possessed distinct expression profiles during differentiation, bound to *Sox6* to influence its effect on satellite cells-derived slow-type fibers. There was a higher expression of slow MyHC in myotubes derived from muscle satellite cells in *Nfix*-null mouse in DM [24], which supported our finding that *Nfix*-low expressed LM-MTs intrinsically generated slow muscles.

Furthermore, *Nfix*-null mice display delayed regeneration and knocking out *Nfix* in satellite cells causes muscle regeneration to be delayed [24,43]. Our results implied a higher self-renewal potential in *Nfix*-low expressed LM-MTs, indicating more satellite cells stay or return to the quiescent stage and less satellite cells participate in muscle repair. These might partially explain the delayed muscle regeneration by *Nfix*-null satellite cells. Moreover, *NFATc4*, which would be downregulated by *Nfix* [44], had been found to improve the pool of reserve cells [45]. Therefore, we argued that a complex regulatory network governs the inherent characteristics in satellite cells.

*Mef2C* had been documented to play a vital role in slow-twitch myofibers [26,46]. Previous studies reported that multiple factors regulated *Mef2C* to control muscle cells [47,48]. Here we showed that *Sox6* activated the transcription of *Mef2C* by directly acting on its proximal promoter, but the presence of *Nfix* inhibited this activation. Moreover, we found that *Mef2C* was able to bind to the slow-type isoforms promoter to promote its transcriptions. These findings implied that the indirect promotion of slow fibers by *Sox6* was due to the expression of *Nfix* in LM-MTs, which was insufficient to interfere with the activation of *Mef2C* by *Sox6*. Previous research demonstrated that *Mef2C* could rescue muscle atrophy [49]. Combined deletion of *MEF2A*, *C* and *D* in mouse hind limb muscles-derived satellite cells prevented the muscle regeneration [50], indicating a positive effect of *Mef2C* on cell self-renewal ability. Hence, the insufficient *Sox6* influence in cell self-renewal potential of LM-MTs might be due to the upregulation of *Mef2C* by *Sox6*. Previous studies have found that the expression of *Sox6* and *Nfix* is closely related to the regulation of fast and slow muscles in skeletal muscle development during embryonic to fetal stages [25,44]. Our results provided a new understanding that *Sox6* could also contribute to the muscle fiber types regulation after birth by interacting with *Nfix* in regulating the myogenesis development of satellite cells.

We also performed functional similarity experiments to validate the roles of *Nfix* and *Mef2C* in the slow fiber regulation of *Sox6*. We found that *Sox6* overexpression in *Nfix*-inhibited PM-MTs promoted slow-type fiber formation, which was consistent with the effects of *Mef2C* overexpression in PM-MTs and *Sox6* overexpression in LM-MTs. In contrast, *Sox6* overexpression in *Nfix*-overexpressing LM-MTs downregulated the slow-type fiber formation, which was consistent with the effect of *Mef2C* inhibition in LM-MTs and *Sox6* overexpression in PM-MTs. These results confirmed that the different effects of *Sox6* on slow fibers were implemented partially through *Mef2C*, which are affected by the expression of *Nfix* in PM-MTs and LM-MTs. A recent study showed that *Sirt6* expression was elevated in chronically exercised humans and *Sirt6* could downregulate *Sox6* by increasing the transcription of CREB to increase the slow-type fibers and exercise endurance, which indicated that *Sirt6* activation may offer an exercise mimetic therapy [51]. Here, whether our research findings about *Sox6* can provide a certain reference for human exercise mimetic therapy remains to be further studied.

## 4. Materials and Methods

### 4.1. Ethics Standards

All animal experimental protocols were carried out in accordance with “The Instructive Notions with Respect to Caring for Laboratory Animals” issued by the Ministry of Science and Technology of the People’s Republic of China and approved by the South China Agricultural University Institutional Animal Care and Use Committee (2022F150).

### 4.2. Isolation of Muscle Satellite Cells and Cell Culture

All chickens were obtained from a single hatch and raised indoors. The chickens were fed a diet containing 2837 kcal ME (metabolic energy)/kg and 200 g CP (crude protein)/kg. Satellite cells were isolated from pectoral and leg muscles of 3-day-old chickens as previously described [52]. Briefly, skeletal muscles were isolated, cut and minced into smooth pulp. The minced tissues were digested with 0.1% collagenase I (Solarbio, Beijing, China) for 30 min at 37 °C. After centrifugation and removing the supernatant, the tissues were digested with 0.25% trypsin (Solarbio, Beijing, China) for 1 h at 37 °C. The digestion was terminated with complete medium containing DMEM/F12 (Invitrogen, Carlsbad, CA, USA), 20% fetal bovine serum (FBS, Gibco, Bethesda, MD, USA), 100 units/mL penicillin and 100 µg/mL streptomycin (final concentration, Gibco, Bethesda, MD, USA). The suspension was transferred onto a cell strainer (70 μm) until it passed through. The cell suspension was centrifuged at 1500 rpm for 8 min then the supernatant was discarded. The cells were resuspended with complete medium and cultured at 37 °C with 100% humidity under 5% CO_2_. After 2 h of culture, the satellite cells were in the cell suspension. Then the cell suspension was transferred to a new cell culture plate and cultured at 37 °C with 100% humidity under 5% CO_2_. The cells were cultured in differentiation medium (DM) containing DMEM/F12, 5% horse serum (Gibco, Bethesda, MD, USA), 100 units/mL penicillin and 100 µg/mL streptomycin to induce myoblast differentiation.

### 4.3. Immunofluorescence

Cultured cells and myotubes were washed with PBS (Solarbio, Beijing, China), fixed with 4% paraformaldehyde (Solarbio, Beijing, China), infiltrated with 0.1% TritonX-100 (Solarbio, Beijing, China), blocked with 5% BSA in PBS containing 5% goat serum (Solarbio, Beijing, China), then stained with the following primary antibodies: anti-Pax7 (D121107, 1:500, Sangon Biotech, Shanghai, China), anti-MyH1A (F59, 1:100, DHSB, Iowa City, IA, USA), anti-MyH7B (S58, 1:100, DHSB), anti-Nfix (2D3, 1:50, DHSB), anti-MyHC (b103, 1:500, DHSB), anti-*Sox6* (bs-21580R, 1:500, Bioss, Beijing, China). Primary antibodies were incubated overnight at 4 °C, then cultures were washed in PBS and incubated with appropriate secondary antibodies conjugated with FITC, RBITC, AlexaFluor 488 or AlexaFluor 555 (all from Sangon Biotech, 1:1000, Shanghai, China) for 1 h at room temperature. The cell nuclei were stained by DAPI (Beyotime, Nantong, China). Fluorescent images were captured by Leica DMi8 fluorescence microscope or Leica TCs SP8 confocal microscope (Leica, Wetzlar, Germany). Image processing and quantification were performed using Leica Las X software (V3.7, Leica, Wetzlar, Germany) or ImageJ software (National Institutes of Health, Bethesda, MD, USA)

### 4.4. Edu and Flow Cytometry Assays

Cell-Light EdU Apollo In Vitro Kit (RiboBio, Guangzhou, China) was used for Edu assay according to the manufacturer’s protocols. Cell nuclei were stained by DAPI. Flow cytometry assays were performed using FxCycle™ PI/RNase Staining Solution (Invitrogen, USA) was used according to the manufacturer’s protocols and analyzed by BD Accuri C6 flow cytometer (BD Biosciences, San Jose, CA, USA).

### 4.5. RNA Extraction, cDNA Synthesis and Quantitative Real-Time PCR

Total RNA was extracted from cultured cells and myotubes using RNAiso plus reagent (Takara, Otsu, Japan) according to the traditional protocols. Complementary DNA (cDNA) synthesis for messenger RNA (mRNA) was carried out using the PrimerScript RT Reagent Kit with gDNA Eraser (Perfect Real Time) (Takara). Quantitative real-time PCR assays were conducted on QuantStudio 5 Real-Time PCR System (Applied Biosystems Inc., Foster, Waltham, MA, USA) using the iTaq Universal SYBR Green Supermix Kit (Bio-Rad Laboratories Inc., Hercules, CA, USA) in triplicate, as described previously [53]. The relative expression levels were calculated using the 2^−^^△△Ct^ relative quantitative method, as described previously [54]. Chicken *β*-actin was used as an internal control. Primers for qRT-qPCR are shown in Table S1.

### 4.6. Plasmid Construction, RNA Oligonucleotides and Cell Transfection

Gene overexpression vectors: pcDNA3.1-*Sox6* for *Sox6* overexpression (NCBI Reference Sequence: NM_001398398.1), pcDNA3.1-Nfix for *Nfix* overexpression (NCBI Reference Sequence: NM_001397397.1), pcDNA3.1-Mef2C for *Mef2C* overexpression (NCBI Reference Sequence: XM_046905690.1), coding sequence were amplified from chicken leg muscle cDNA by PCR and ligated into the pcDNA3.1 vector (Invitrogen). The successful overexpression vector was confirmed by DNA sequencing. Primers for coding sequence cloning are shown in Table S2.

Promoter reporter plasmid: different lengths of *Mef2C*, *MyH1A*, *Tnnc2*, *Tnni2*, *Tnnt3*, *MyH7B* and *Tnnc1* promoter sequences were amplified from chicken leg muscle DNA by PCR. The PCR products were isolated from agarose gel and ligated into the pGL3-basic luciferase reporter vector (Promega, Madison, WI, USA). Primers for promoter sequence cloning are shown in Table S2.

Small interfering RNA (siRNA) against *Sox6*, *Nfix* and *Mef2C* were designed and synthesized by Ribobio (Table S3, Guangzhou, China), nonspecific duplex siNC was used as control and provided by Ribobio. Cell transfection was carried out using Lipofectamine 3000 reagent (Invitrogen) according to the manufacturer’s protocols. Lipofectamine 3000 reagents and nucleic acids were diluted in OPTI-MEM with Reduced Serum Medium (Gibco).

### 4.7. Western Blot

Total protein from cultured myotubes was extracted using RIPA lysis buffer (Beyotime) with PMSF protease inhibitor (Beyotime), after incubation for 15 min on ice and centrifugation at 13,000× *g* for 10 min at 4 °C, the supernatant was collected. The proteins were separated in SDS-PAGE gels and transferred into polyvinylidene fluoride membranes (Bio-Rad Laboratories Inc.), subsequently blocked in QuickBlock™ Western blocking buffer (Beyotime). The membranes were then incubated overnight at 4 °C with primary antibodies: anti-MyH1A (F59, DHSB, 1:100), anti-MyH7B (S58, DHSB, 1:100), anti-Nfix (2D3, DHSB, 1:50), anti-*β*-actin (AF5003, Beyotime, 1:1000). Then membranes were washed in PBS and incubated with appropriate secondary antibodies conjugated with Dylight 680 (BS10037, BS10038, Bioworld, Nanjing, China, 1:1000) for 1 h at room temperature. The blots were developed using the Odyssey Fc imaging system (LI-COR, NE, USA).

### 4.8. Dual-Luciferase Reporter Assay

The chicken embryonic fibroblast cell line (DF-1) was maintained in medium containing Dulbecco’s modified Eagle’s medium (Invitrogen) with high glucose, 10% fetal bovine serum (Gibco), 100 units/mL penicillin and 100 µg/mL streptomycin (final concentration, Gibco) and incubated at 37 °C with 100% humidity under 5% CO_2_. For essential promoter validation, the DF-1 cells were transfected with a series of the promoter reporter plasmids described above and the TK-Renilla reporter (Promega) was co-transfected as internal control. For interaction assays, the promoter reporter plasmids were co-transfected with the gene overexpression vectors in DF-1 and the TK-Renilla reporter (Promega) was co-transfected as internal control. At 48 h after transfection, the luciferase activities and Renilla luciferase of the cells were measured using the Dual-Glo^®^ Luciferase Assay System (Promega) and Synergy™ Neo 2 Multi-Mode Microplate Reader (Biotek, Winooski, VT, USA) with Gen5 software (Biotek, Winooski, VT, USA). The levels of firefly luciferase activity were normalized to Renilla luciferase activity.

### 4.9. Co-Immunoprecipitation Assay

Co-immunoprecipitation assays were performed using Pierce Co-Immunoprecipitation (Co-IP) Kit (Thermo Scientific, MA, USA) in accordance with the manufacturer’s protocols. Briefly, cultured cells were washed by 1× Modified Dulbecco’s PBS and incubated with ice-cold IP Lysis/Wash Buffer on ice for 5 min with periodic mixing. Lysates were centrifuged at 13,000× *g* for 10 min to pelletize the cell debris. Antibody immobilizations were prepared by adding appropriate amount of AminoLink Plus Coupling Resin, Coupling Buffer, Sodium Cyanoborohydride Solution, ultrapure water and 75 µg of anti-*Sox6* or IgG antibody (bs-0310G, Bioss, Beijing, China) to a tube and incubating on a mixer at room temperature for 120 min. For immunoprecipitation, protein lysates and antibodies-binding resin were mixed and incubated with gentle mixing at 4 °C overnight. After centrifugation and removing the flow-through, the mixture was washed with cold IP Lysis/Wash Buffer and the antibody-binding protein complexes were eluted from the resin mixture using 50 µL Elution Buffer. Proteins were denatured by adding 12.5 µL 5× SDS-PAGE Sample Buffer and boiling at 100 °C for 5 min, then applied to SDS-PAGE analysis.

### 4.10. Statistical Analysis

The statistical analyses were performed using the two-tailed Student’s *t*-test for comparing two groups, or the one-way analysis of variance with Tukey’s multiple comparison test for comparing among multiple groups. All data were presented as mean values with error bars representing standard error of mean. Probability of less than 5% (*p* < 0.05) was considered to be statistically significant.

## 5. Conclusions

**Figure 9:**
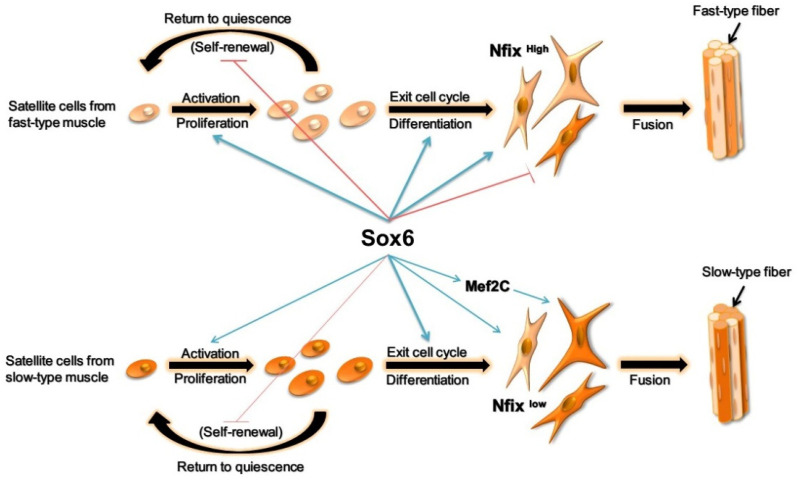
The regulation mechanism of *Sox6* in satellite cells from fast-type and slow-type muscles. In brief, *Sox6* promotes the proliferation and differentiation of PM-MBs and LM-MBs, improves the fast-type fiber formation. The upregulation of *Nfix* in PM-MTs inhibits the slow-type fiber formation via the collaboration with *Sox6*. In LM-MTs, *Sox6* activates *Mef2C* transcription to promote the slow-type fiber formation.

In conclusion, we demonstrated that the satellite cell populations from different muscle fiber types are heterogeneous and they possessed an intrinsically predetermined property to generate specific muscle fiber types similar to their original muscle. Furthermore, *Sox6* could directly promote the fast fiber isoform transcriptions and the different effects of *Sox6* on slow-type fibers were due to the distinct expression patterns of *Nfix* between PM-MTs and LM-MTs. Highly expressed Nfix in PM-MTs was bound with *Sox6* to form a complex, inhibiting slow-type fiber formation. However, *Nfix* in LM-MTs was insufficient to exhibit the same role, resulting in the *Mef2C* activation by *Sox6* and increased regulation of slow fiber isoforms by *Mef2C* (Figure 9). These findings could partially explain the myogenic differences of satellite cells from different muscles, helping in broadening the understanding of molecular mechanisms involved in the satellite cell myogenesis and chicken skeletal muscle development.

## Data Availability

All data generated or analyzed during this study are included in this published article and its additional file.

## Supplementary Materials

The following supporting information can be downloaded at: https://www.mdpi.com/article/10.3390/ijms231911327/s1.
