# Supplementary material for: Sox6 Differentially Regulates Inherited Myogenic Abilities and Muscle Fiber Types of Satellite Cells Derived from Fast- and Slow-Type Muscles
Source: Int J Mol Sci. 2022 Sep 26;23(19):11327. doi: 10.3390/ijms231911327 (PMC9569562; doi:10.3390/ijms231911327)
Supplement: Supplementary file 1 [file ijms-23-11327-s001.zip › Supplementary Figures.pdf]

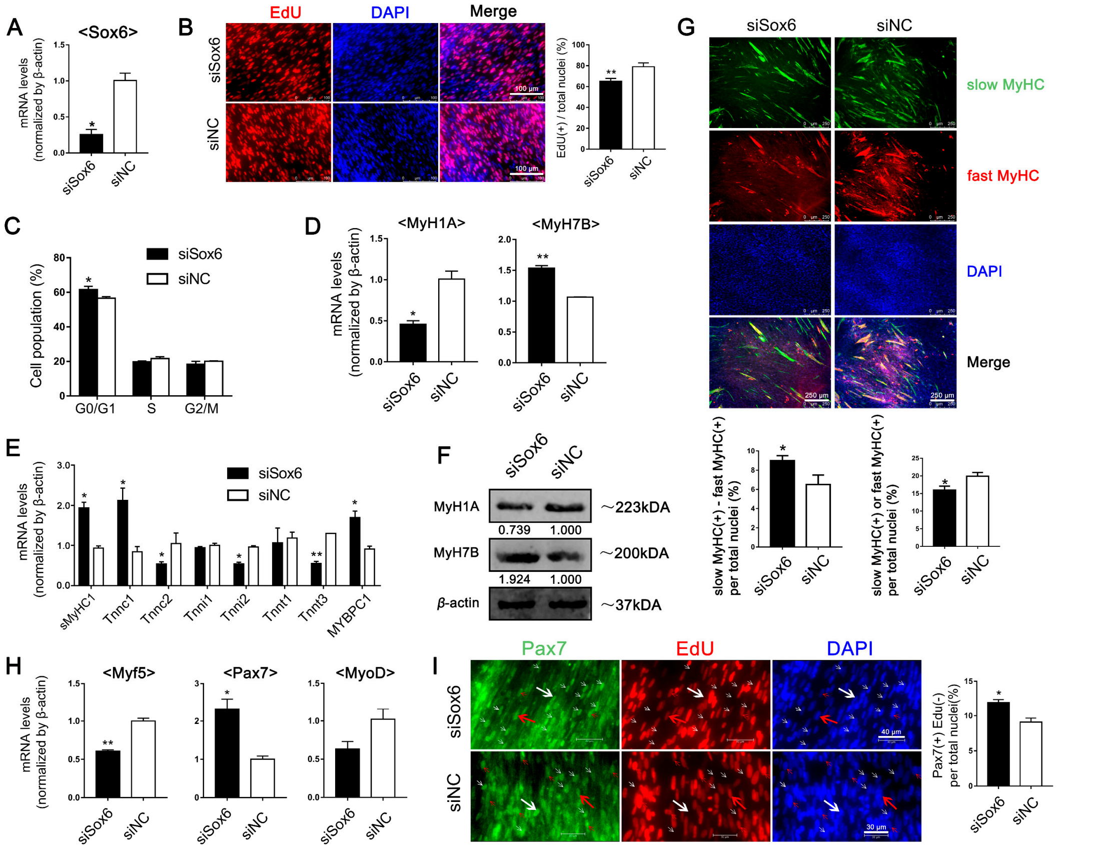

Figure S1. Inhibition of *Sox6* in PM-derived satellite cell inhibits cell proliferation and fast-type fiber formation, but improves slow-type fiber formation and cell self-renewal potential. (**A**) The relative expression levels of *Sox6* in *Sox6*-inhibited PM-MBs were quantified by qPCR (mean ± SEM; * $p < 0.05$; $n = 3$; two-tailed Student's t-test). (**B**) Proliferation of *Sox6*-inhibited PM-MBs were assessed by Edu (mean ± SEM; ** $p < 0.01$; $n = 3$; two-tailed Student's t-test). (**C**) Cell cycle analysis of *Sox6*-inhibited PM-MBs (mean ± SEM; * $p < 0.05$; $n = 3$; two-tailed Student's t-test). (**D**) After inducing differentiation for 3 days, the relative expression levels of *MyH1A* and *MyH7B* in *Sox6*-inhibited PM-MTs were quantified by qPCR (mean ± SEM; * $p < 0.05$; ** $p < 0.01$; $n = 3$; two-tailed Student's t-test). (**E**) After inducing differentiation for 3 days, the relative expression levels of fast- and slow-type fiber isoforms in *Sox6*-inhibited PM-MTs were quantified by qPCR (mean ± SEM; * $p < 0.05$; ** $p < 0.01$; $n = 3$; two-tailed Student's t-test). (**F**) Western blot on lysates from *Sox6*-inhibited PM-MTs and control groups myotubes after inducing differentiation for 3 days. *β*-actin was used to normalize. (**G**) After inducing differentiation for 3 days, myotubes derived from *Sox6*-inhibited PM-MTs were stained against S58 (slow-type MyHC, green) and F59 (fast-type MyHC, red) and nuclei were counterstained with DAPI. The slow MyHC(+) nuclei or fast MyHC(+) nuclei were counted. The proportion of slow MyHC(+) cells − fast MyHC(+) cells among total nuclei and the proportion of cells in slow MyHC(+) myotubes or fast MyHC(+) myotubes among total nuclei are presented as mean ± SEM (* $p < 0.05$; $n = 3$; two-tailed Student's t-test). (**H**) After inducing differentiation for 3 days, the relative expression levels of *Myf5*, *Pax7* and *MyoD* in *Sox6*-inhibited PM-MBs were quantified by qPCR (mean ± SEM; * $p < 0.05$; ** $p < 0.01$; $n = 3$; two-tailed Student's t-test). (**I**) *Sox6*-inhibited PM-MBs were cultured in DM for 5 days and EdU was added to the culture medium 24 hours prior to harvest. In the end, the cells were stained against Pax7 (green) and EdU (red) and the numbers were counted (mean ± SEM; * $p < 0.05$; $n = 3$; two-tailed Student's t-test). White arrows represented part of the typical Pax7(+)EdU(-) cells and red arrows represented part of the typical Pax7(+)EdU(+) cells.

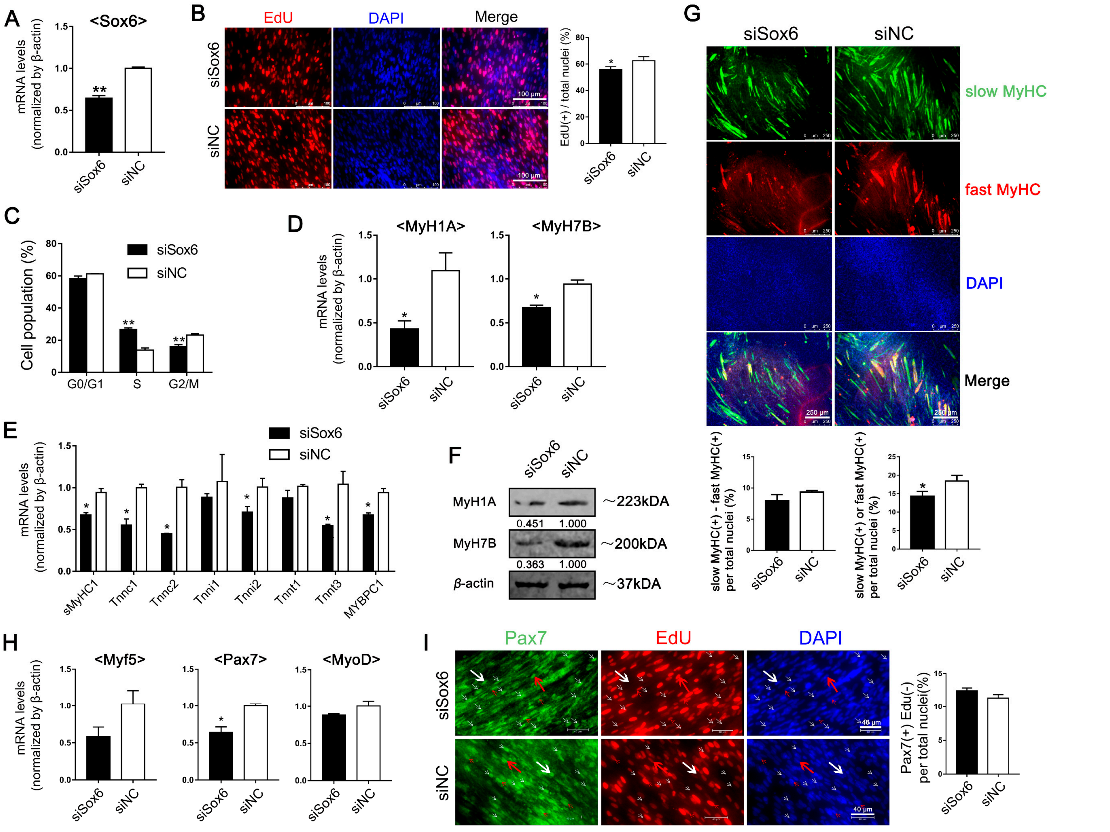

Figure S2. Inhibition of *Sox6* in LM-derived satellite cells inhibits cell proliferation and fast-type, slow-type fibers formation. (**A**) The relative expression levels of *Sox6* in *Sox6*-inhibited LM-MBs were quantified by qPCR (mean ± SEM; ** $p < 0.01$; $n = 3$; two-tailed Student's t-test). (**B**) Proliferation of *Sox6*-inhibited LM-MBs were assessed by Edu (mean ± SEM; * $p < 0.05$; $n = 3$; two-tailed Student's t-test). (**C**) Cell cycle analysis of *Sox6*-inhibited LM-MBs (mean ± SEM; ** $p < 0.01$; $n = 3$; two-tailed Student's t-test). (**D**) After inducing differentiation for 3 days, the relative expression levels of *MyH1A* and *MyH7B* in *Sox6*-inhibited LM-MTs were quantified by qPCR (mean ± SEM; * $p < 0.05$; $n = 3$; two-tailed Student's t-test). (**E**) After inducing differentiation for 3 days, the relative expression levels of fast- and slow-type fiber isoforms in *Sox6*-inhibited LM-MTs were quantified by qPCR (mean ± SEM; * $p < 0.05$; $n = 3$; two-tailed Student's t-test). (**F**) Western blot on lysates from *Sox6*-inhibited LM-MTs and control groups myotubes after inducing differentiation for 3 days. *β*-actin was used to normalize. (**G**) After inducing differentiation for 3 days, Myotubes derived from *Sox6*-inhibited LM-MTs were stained against S58 (slow-type MyHC, green) and F59 (fast-type MyHC, red) and nuclei were counterstained with DAPI. The proportion of slow MyHC(+) cells − fast MyHC(+) cells among total nuclei and the proportion of cells in slow MyHC(+) myotubes or fast MyHC(+) myotubes among total nuclei are presented as mean ± SEM (* $p < 0.05$; $n = 3$; two-tailed Student's t-test). (**H**) After inducing differentiation for 3 days, the relative expression levels of *Myf5*, *Pax7* and *MyoD* in *Sox6*-inhibited LM-MBs were quantified by qPCR (mean ± SEM; * $p < 0.05$; $n = 3$; two-tailed Student's t-test). (**I**) *Sox6*-inhibited LM-MBs were cultured in DM for 5 days and EdU was added to the culture medium 24 hours prior to harvest. In the end, the cells were stained against Pax7 (green) and EdU (red) and the numbers were counted (mean ± SEM; $n = 3$; two-tailed Student's t-test). White arrows represented part of the typical Pax7(+)EdU(-) cells and red arrows represented part of the typical Pax7(+)EdU(+) cells.

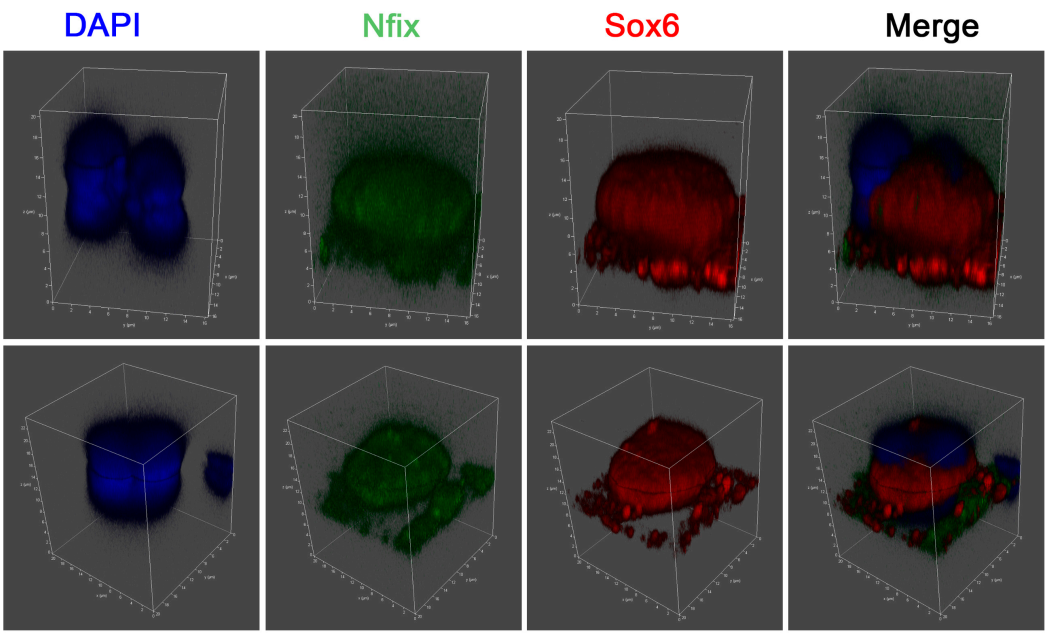

Figure S3. Multiple myonuclei fusion in the co-localization of Sox6 and Nfix. Myotubes derived from PM-MBs were performing immunofluorescence double staining against Sox6 (red) and Nfix (green). The three-dimensional view of Sox6 and Nfix localization in PM-MTs was presented through confocal microscopy.

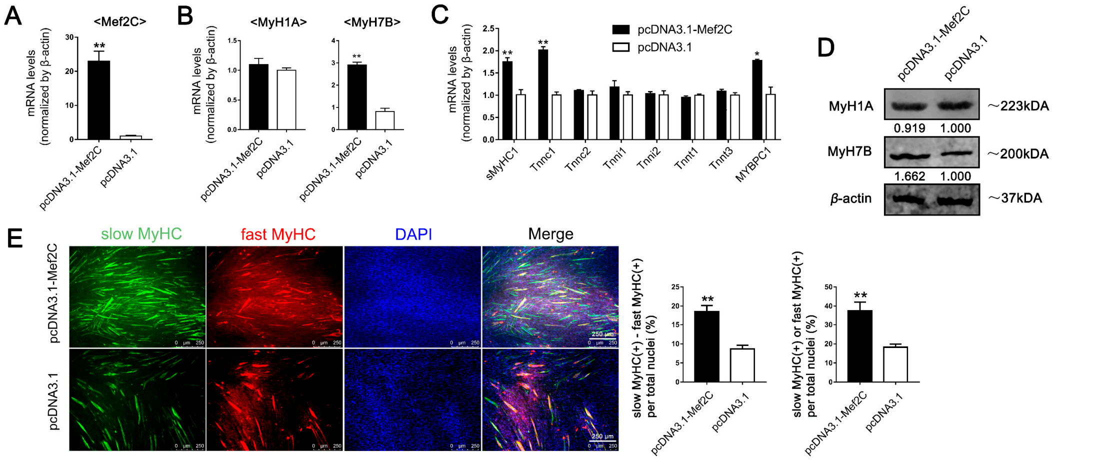

Figure S4. Overexpression of *Mef2C* in PM-MTs promotes slow-type fiber formation. (**A**) The relative expression levels of *Mef2C* in *Mef2C*-overexpressing PM-MTs was quantified by qPCR (mean ± SEM; ** $p < 0.01$; $n = 3$; two-tailed Student's t-test). (**B**) After inducing differentiation for 3 days, the relative expression levels of *MyH1A* and *MyH7B* in *Mef2C*-overexpressing PM-MTs were quantified by qPCR (mean ± SEM; ** $p < 0.01$; $n = 3$; two-tailed Student's t-test). (**C**) After inducing differentiation for 3 days, the relative expression levels of fast- and slow-type fiber isoforms in *Mef2C*-overexpressing PM-MTs were quantified by qPCR (mean ± SEM; * $p < 0.05$; **$p < 0.01$; $n = 3$; two-tailed Student's t-test). (**D**) Western blot on lysates from *Mef2C*-overexpressing PM-MTs and control group myotubes after inducing differentiation for 3 days. *β*-actin was used to normalize. (**E**) After inducing differentiation for 3 days, myotubes derived from *Mef2C*-overexpressing PM-MTs were performing immunofluorescence double staining against S58 (slow-type MyHC, green) and F59 (fast-type MyHC, red) and nuclei were counterstained with DAPI. The slow MyHC(+) nuclei or fast MyHC(+) nuclei were counted. The proportion of slow MyHC(+) cells − fast MyHC(+) cells among total nuclei and the proportion of cells in slow MyHC(+) myotubes or fast MyHC(+) myotubes among total nuclei are presented as mean ± SEM (** $p < 0.01$; $n = 3$; two-tailed Student's t-test).

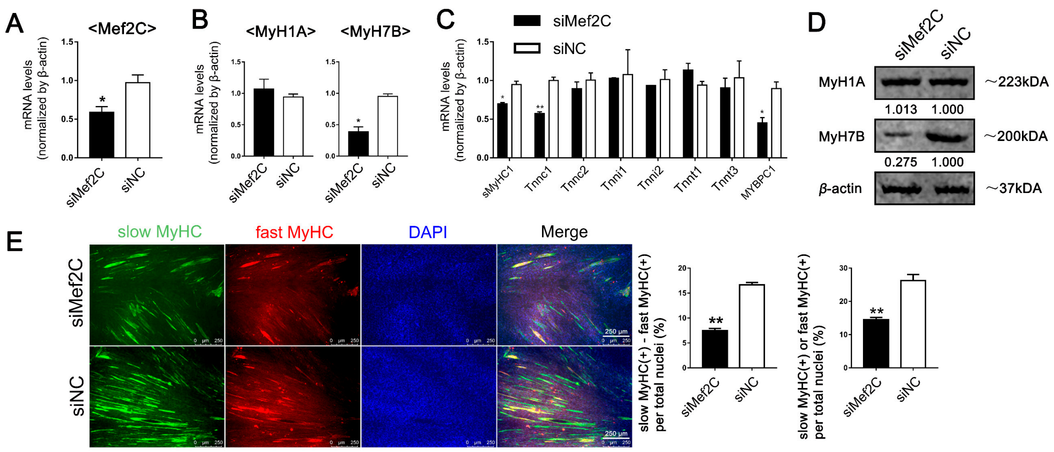

Figure S5. Inhibition of *Mef2C* in LM-MTs decreases slow-type fiber formation. (**A**) The relative expression levels of *Mef2C* in *Mef2C*-inhibited LM-MTs was quantified by qPCR (mean ± SEM; * $p < 0.05$; $n = 3$; two-tailed Student's t-test). (**B**) After inducing differentiation for 3 days, the relative expression levels of *MyH1A* and *MyH7B* in *Mef2C*-inhibited LM-MTs were quantified by qPCR (mean ± SEM; * $p < 0.05$; $n = 3$; two-tailed Student's t-test). (**C**) After inducing differentiation for 3 days, the relative expression levels of fast- and slow-type fiber isoforms in *Mef2C*-inhibited LM-MTs were quantified by qPCR (mean ± SEM; * $p < 0.05$; ** $p < 0.01$; $n = 3$; two-tailed Student's t-test). (**D**) Western blot on lysates from *Mef2C*-inhibited LM-MTs and control group myotubes after inducing differentiation for 3 days. *β*-actin was used to normalize. (**E**) After inducing differentiation for 3 days, myotubes derived from *Mef2C*-inhibited LM-MTs were performing immunofluorescence double staining against S58 (slow-type MyHC, green) and F59 (fast-type MyHC, red) and nuclei were counterstained with DAPI. The slow MyHC(+) nuclei or fast MyHC(+) nuclei were counted. The proportion of slow MyHC(+) cells − fast MyHC(+) cells among total nuclei and the proportion of cells in slow MyHC(+) myotubes or fast MyHC(+) myotubes among total nuclei are presented as mean ± SEM (** $p < 0.01$; $n = 3$; two-tailed Student's t-test).
