# Supplementary material for: Sox6 Differentially Regulates Inherited Myogenic Abilities and Muscle Fiber Types of Satellite Cells Derived from Fast- and Slow-Type Muscles
Source: Int J Mol Sci. 2022 Sep 26;23(19):11327. doi: 10.3390/ijms231911327 (PMC9569562; doi:10.3390/ijms231911327)
Supplement: Supplementary file 1 [file ijms-23-11327-s001.zip › Supplementary Tables.pdf]

SUPPLEMENTARY MATERIALS

Supplementary Table S1. Primers used for qRT-PCR

**Gene**	**Primers**	**Sequence (5' to3')**
Pax7	Forward	TCAGCAACCGACGAGCAAGATG
	Reverse	TGGTTGGGTAGGTGGAGTCTGG
MyH1A	Forward	CTGGAGGAGAAAATGGTGTCCCTG
	Reverse	CCCGTTCTGTCACCTCCTTAATTT
MyH7B	Forward	CCAGCTACAGGCAGAGCAAGACAA
	Reverse	TTCATCTCCTCCTCGTCCTCCACA
Sox6	Forward	AGCCTGGCAGAAAAAGAGCGAC
	Reverse	AGGCATGTGACCCTGAACCTGAA
sMyHC1	Forward	CCATGCCGAGAGCGTCAAA
	Reverse	CTTGGACAGGTTGCAGTTGGAC
Tnnc1	Forward	GAGGTGGATGAGGATGGCAGT
	Reverse	TGATCGTCTCTCCAGTTGCCTG
Tnnc2	Forward	GCCAGATGAAAGAGGACGCCAAGG
	Reverse	CTGCACACCCTCCATCATCTTCAG
Tnni1	Forward	GACCTGAAGCTCAAAGTGCTCGAC
	Reverse	CTCCACGTTCTTGCGCCAATCACC
Tnni2	Forward	GGATGAGGAAAGGTATGACACAGAGG
	Reverse	CCTTCTTGACTTGCTTCAGGTTGG
Tnnt1	Forward	GCCGAGGATGACGCCAAGAAGAAG
	Reverse	CGCAGCTTCTCCATCAGGTCGAAC
Tnnt3	Forward	GAAAGAGCAGAGCAACAGAGAATCC
	Reverse	GTTTGCTTCTTCCCTCTCTTCTGA
MYBPC1	Forward	CTGCTGTTGATGATGAGGGTGAATAC
	Reverse	GTGTTATTTTCCCCAAGACCATCAAG
Myf5	Forward	TTGAGGGAACAGGTGGAGAACTAC
	Reverse	GAGAGAGGCGGTCCACGATG
MyoD	Forward	CAACGCCATCCGCTACATCG
	Reverse	TTTGGGTCATTTGGTGATTCCGT
Nfix	Forward	CGGACAATCCGACAGCTCCA
	Reverse	TAGCCACAGGGGTCTGGGAC
Mef2C	Forward	AGCCGCATGAGAGTCGAAC
	Reverse	TGAATCGTCTGCATCGGGG
β-actin	Forward	CCCCACGCCATCCTCCGTCTG
	Reverse	CCTCGGGGCACCTGAACCTCTC

Supplementary Table S2. Primers used for cloning

Plasmids	Primer	Sequence (5' to3')
pcDNA3.1-Sox6	F	CGG**GGTACC**ATGTCTTCCAAGCAGGCTA
	R	CCG**GAATTC**TCAGTTGGCACTGACTG
pcDNA3.1-Nfix	F	CGG**GGTACC**ATGTACTCCCCGTATTGCCTTACCCA
	R	CCG**GAATTC**TCAGAAAGTTGCTGTCGCGGGGTCC
pcDNA3.1-Mef2C	F	CCC**AAGCTT**ATGGGGAGAAAAAAGATTCAGATTAC
	R	CGC**GGATCC**TCATGTCGCCCATCCTTCAG
pGL3-Mef2C-1.5kb	-1564	CGG**GGTACC**TGCCTAAGTCTGAAGGTAATGCAAAC
pGL3-Mef2C-1.1kb	-1108	CGG**GGTACC**GCTCCTGACCCACATTTTTCTCATCAT
pGL3-Mef2C-0.7kb	-746	CGG**GGTACCC**CAAAAAGGACAAGAAGACCAAGTTTAG
pGL3-Mef2C-0.4kb	-403	CGG**GGTACCC**CAAAAAGGACAAGAAGACCAAGTTTAG
Common antisense	-29	CCC**AAGCTT**GTTTGCCTCACTCACCCTCTCC
pGL3-MyH1A-2.3kb	-2317	CGG**GGTACC**GACATCCTGTAGCAGACAGACTATA
pGL3-MyH1A-1.8kb	-1823	CGG**GGTACC**ACCCCTCTGCTTCCATCTGTCATAC
pGL3-MyH1A-1.2kb	-1260	CGG**GGTACC**TCTGAAAATTGACAGGGAATGTA
pGL3-MyH1A-0.7kb	-720	CGG**GGTACC**AAGTGATAAAATCAAATGAAGAAGCA
Common antisense	-1	CCC**AAGCTT**AGGACAGCACAGCTTCAAGGAATC
pGL3-Tnnc2-1.2kb	-1206	CGG**GGTACC**CCAACACCACGACATCCCAAC
pGL3-Tnnc2-0.8kb	-796	CGG**GGTACC**GCATCATTCCCACTCTCCCAG
pGL3-Tnnc2-0.3kb	-319	CGG**GGTACC**GACAAGGAGGCGTAGGTGAAG
Common antisense	+4	CCC**AAGCTT**CCATCTTTGCTGCTCTCCTCC
pGL3-Tnni2-1.6kb	-1665	CGG**GGTACC**CGCTTGTCACCGTTTGTCTGCTG
pGL3-Tnni2-1.1kb	-1109	CGG**GGTACC**ACCTAAACTACATCTCCCAGGC
pGL3-Tnni2-0.6kb	-599	CGG**GGTACC**CTGACCCCTGTGTGTGGTTTCT
Common antisense	+31	CCG**CTCGAG**GGTCCAATTCCCCTGCAATGAGC
pGL3-Tnnt3-1.0kb	-1051	CGG**GGTACC**GAGCCAAAAATATCACTTGTCCTG
pGL3-Tnnt3-0.5kb	-589	CGG**GGTACC**GTCTCCTTCAATGCTAATGCTC
Common antisense	-1	CCG**CTCGAG**GCTCTCAAGTTATAGTGACATTCG
pGL3-MyH7B-1.3kb	-1386	CGG**GGTACC**GGATTTGGGCATTTAGTGATGGACAGA
pGL3-MyH7B-0.9kb	-909	CGG**GGTACC**GTGTGGACATGACACATGGGAAT
pGL3-MyH7B-0.4kb	-416	CGG**GGTACC**CTTTGCAGAGGCTTTGTGTAGAGCA
Common antisense	-1	CCC**AAGCTT**CTTGTCTGGCTGGAGGAGGACTTG
pGL3-Tnnc1-1.6kb	-1635	CGG**GGTACC**GGAGAAACGCGCAGCCGCCTTCA
pGL3-Tnnc1-0.9kb	-972	CGG**GGTACC**GCCTTTGTGCCTCTGCCGTGTCCC
pGL3-Tnnc1-0.4kb	-419	CGG**GGTACC**GACGCTCCCGATTTGCCCCTGCC
Common antisense	+17	CCC**AAGCTT**GCCCCGCTGCCAACATCTCCTCCT

The nucleotide positions of primers are shown according to their location relative to the first nucleotide of the first exon or intron of the genes. Restriction endonuclease sequences are shown in bold.

Supplementary Table S3. Small interfering RNA (siRNA)

**Primers**	**Sequence (5' to3')**
siNC	RiboBio supplied
siSox6	GCAGAAGGAAGTAAAGCAA (Target Sequence)
siNfix	TCAAGGAGCTCGATCTCTA (Target Sequence)
siMef2C	GCAGTCTGGTATACAGTAA (Target Sequence)
